# Phytochemical Analysis of *Anvillea garcinii* Leaves: Identification of Garcinamines F–H and Their Antiproliferative Activities

**DOI:** 10.3390/plants10061130

**Published:** 2021-06-02

**Authors:** Hanan Y. Aati, Shagufta Perveen, Raha Orfali, Areej M. Al-Taweel, Jiangnan Peng, Sobia Tabassum, Maged S. Abdel-Kader, Hasan Soliman Yusufoglu, Orazio Taglialatela-Scafati

**Affiliations:** 1 Department of Pharmacognosy, College of Pharmacy, King Saud University, P.O. Box 22452, Riyadh 11495, Saudi Arabia; hati@KSU.EDU.SA (H.Y.A.); rorfali@ksu.edu.sa (R.O.); amaltaweel@KSU.EDU.SA (A.M.A.-T.); 2 Department of Chemistry, School of Computer, Mathematical and Natural Sciences, Morgan State University, Baltimore, MD 21251, USA; jiangnan.peng@morgan.edu; 3 Interdisciplinary Research Centre in Biomedical Materials (IRCBM), Lahore Campus, COMSATS University Islamabad, Islamabad 44000, Pakistan; sobiatabassum@cuilahore.edu.pk; 4 Department of Pharmacognosy, College of Pharmacy, Prince Sattam Bin Abdulaziz University, P.O. Box 173, Al-Kharj 11942, Saudi Arabia; mpharm101@hotmail.com (M.S.A.-K.); h.yusufoglu@psau.edu.sa (H.S.Y.); 5 Department of Pharmacy, School of Medicine and Surgery, University of Naples Federico II, Via Montesano 49, 80131 Naples, Italy; scatagli@unina.it

**Keywords:** *Anvillea garcinii*, medicinal plants, sesquiterpenoids, amino acid, structure elucidation, antiproliferative activity

## Abstract

*Anvillea garcinii* is a medicinal plant used in the Arab region for intestinal diseases, lung and liver diseases, digestive problems, and as an antidiabetic agent. Repeated chromatographic purifications of *A. garcinii* leaves led to the isolation of three undescribed guaiane sesquiterpene derivatives, named garcinamines F–H, characterized by the presence of an amino acid unit, along with five known sesquiterpene lactones (garcinamines B–E and 9β-hydroxyparthenolide). The structures of the new compounds were established using spectroscopic (1D and 2D NMR) and spectrometric methods (ESIMS). Garcinamine H possesses a double bond at the Δ^1,10^ position, a structural feature rarely reported in guaianolide-type sesquiterpenes. The antiproliferative activity of the isolated sesquiterpenes was screened against three different cancer cell lines, and 9β-hydroxyparthenolide and garcinamines C and D displayed significant effects against lung carcinoma (A549), colon carcinoma (LoVo), and breast carcinoma (MCF7) cell lines.

## 1. Introduction

Cancer is the second-most life-threatening disease, with a worldwide incidence leading to nearly 9.5 million deaths and 18 million cases in 2018, with an expected doubling of cases and deaths by 2050 [1,2]. The Arabian Peninsula makes no exception to these general figures: it has been found that the number of known cancer cases increased by 136% from 1999 to 2015 and is projected to rise by a further 63% in 2030. Breast, colorectal and thyroid cancers, and non-Hodgkin’s lymphoma accounted for the largest contributions to these increased rates [3]. Cancer therapy faces numerous challenges, since most chemotherapeutic drugs are not fully effective and are often associated with many unpleasant side effects such as myelosuppression, peripheral neuropathy, and leukopenia [4,5]. Since about 83% of non-biological anticancer drugs are related to natural products [6], researchers have been investigating the possible additions of plant extracts or pure phytochemicals to enhance their therapeutic potency or decrease the adverse effects [7,8].

*Anvillea garcinii* (Burm.f.) DC. (family Asteraceae) is one of the distinctive ethnomedicinal plants used in the Arabian Peninsula to cure many illnesses, such as cold, digestive problems, pulmonary infections, and liver diseases [9]. *A. garcinii* is a small woody shrub that is densely branched with triangular leaves, is green-grey in color, and has yellow flowers. It usually flowers in spring but can also flower at other times in the year. The predominant classes of secondary metabolites characterized by previous phytochemical investigations carried on this plant were flavonoids and sesquiterpene lactones [10,11] that most likely work synergistically to achieve the interesting biological activities exerted by *A. garcinii*. Our previous investigations on *A. garcinii* specimens growing in Saudi Arabia revealed the presence of sesquiterpene lactones of the guaianolide and germacranolide types, as well as of flavonoid glycosides [12,13,14,15]. 

During the last decades, sesquiterpene lactones have attracted wide attention because of their multi-faceted biological activities, including antitumor and anti-inflammatory properties [16]. In several cases, these activities have been rationalized based on the presence of the α-methylene-γ-butyrolactone moiety in their structures, which could act as an electrophilic agent in Michael-type reactions, especially with free cysteine residues. The biological activities could derive from the reversible or irreversible inhibition of enzymes and other functional proteins [16]. It should be noted that the presence of the electrophilic moiety is not sufficient to completely describe the activity, since in many cases the sesquiterpene skeleton plays a crucial modulation role. For example, guaianolides (sesquiterpenes with a 5/7 ring architecture) have been shown in some cases to possess a higher antiproliferative activity compared to other classes of sesquiterpene lactones [17].

Herein we report the result of a phytochemical characterization of the sesquiterpene profile of a specimen of *A. garcinii* and the evaluation of the antiproliferative activity of the isolated compounds on lung carcinoma (A549), colon carcinoma (LoVo), and breast carcinoma (MCF7) cell lines, which are three of the most represented human cancers.

## 2. Results and Discussion

**Figure 1:**
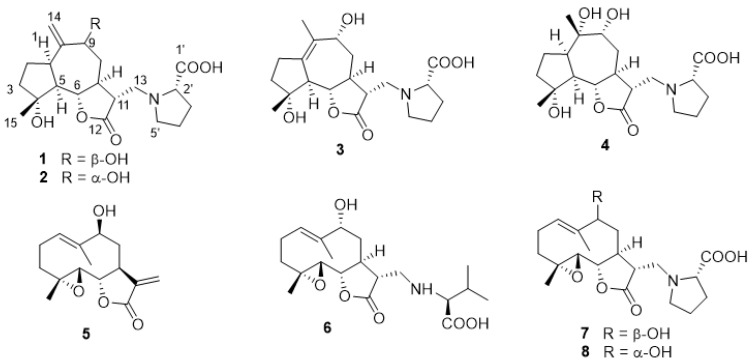
The sesquiterpenoid derivatives isolated from *A. garcinii*.

Previous investigations on the aerial parts of *A. garcinii* [12,13,14,15] identified the *n*-butanol fraction of the ethanol extract as the richest in polar sesquiterpenoids. Its chromatographic separation was achieved using a combination of Sephadex LH-20, silica gel, and RP-18 column chromatography, and yielded three new (**1**–**3**) and five known compounds (**4**–**8**) (Figure 1). The structures of these metabolites were elucidated by spectroscopic analysis, mainly one-dimensional (1D) and two-dimensional (2D) NMR spectroscopy and electrospray ionization mass spectrometry (ESI-MS) (Figure S1–S18). Compounds **4**–**8** were identified as garcinamine E (**4**), 9β-hydroxyparthenolide (**5**), garcinamine B (**6**), garcinamine D (**7**), and garcinamine C (**8**) by comparison of their spectroscopic data with those reported in the literature [12,13,14,15]. 

### 2.1. Structural Elucidation of Garcinamines F–H *(**1**–**3**)*

Garcinamine F (**1**) was obtained as an optically active yellow gum with the molecular formula C_20_H_28_NO_6_, established by ESI-MS (negative ions). The ^13^C NMR and DEPT spectra of **1** (see Supplementary Materials) showed twenty carbon atoms, encompassing only one methyl group (δ_C_ 22.5), eight methylene carbons (seven *sp*^3^ and one *sp*^2^ at δ_C_ 108.5), seven *sp*^3^ methines, and four unprotonated carbon signals, including an ester carbonyl (δ_C_ 171.9) and a carboxylic acid (δ_C_ 177.0) (Table 1). 

**Figure 2:**
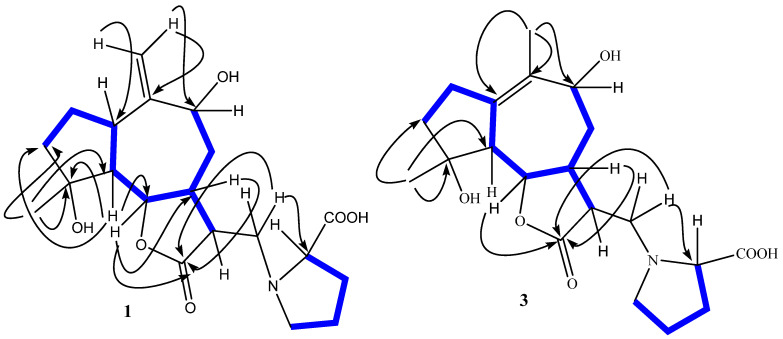
Selected HMBC (→) and COSY (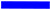) NMR correlations of garcinamines F (**1**) and H (**3**).

The ^1^H NMR spectrum of garcinamine F (Table 2) showed signals for a methyl singlet (δ_H_ 1.33), two exomethylene *sp*^2^ protons (δ_H_ 5.17 and 5.51), two oxymethine protons (δ_H_ 3.99 and 4.23), and two downfield methylene protons (δ_H_ 3.53/3.63 and 3.25/3.86). The COSY spectrum allowed the organization of all the multiplets of the ^1^H NMR spectrum within a large spin system (Figure 2), which was strongly reminiscent of a guaianolide-type sesquiterpene lactone based on comparison with data of related compounds from the same source [12,13,14,15]. The ^1^H NMR spectra also showed typical signals of an *L*-proline moiety including the nitrogen-linking methine at δ_H_ 4.02 and methylene at δ_H_ 3.25 and 3.86. The planar structure of garcinamine F was then unambiguously defined based on the long-range H/C correlations evidenced by the 2D HMBC NMR spectrum (Figure 2).

The key HMBC correlations were those from H_3_-15 to C-3, C-4, and C-5; from H_2_-14 to C-1, C-9, and C-10; from H-6 to C-12; and from H_2_-13 to the proline carbons C-2′ and C-5′. ROESY correlations of H-6 with H_3_-15 and H-11, of H-5 with H-1 and H-7, and of H-7 with H-9 defined the relative configuration of the stereogenic centers.

Garcinamine G (**2**) showed the same molecular formula C_20_H_28_NO_6_ as garcinamine F and also very similar ^1^H (Table 2) and ^13^C (Table 1) NMR spectra (see Supplementary Materials). Analysis of COSY, HSQC, and HMBC 2D NMR spectra led to the assignment of all the H/C resonances and to the definition of a planar structure identical to that of garcinamine F, clearly pointing to a diastereomeric relationship between the two compounds. The most significant differences in the ^13^C NMR spectra of garcinamines F and G were confined to the signals of C-8, C-9, C-10, and C-14. Similarly, the ^1^H NMR spectra of these two compounds mainly differed for the resonances of the seven-membered ring protons. These spectral data strongly suggest that garcinamine G (**2**) should be the 9-epimer of garcinamine F. The marked change in the coupling constant pattern of H-9 and the ROESY cross-peaks of H-6 and H-9 with H-8β support this conclusion.

Additionally, garcinamine H (**3**) showed the molecular formula C_20_H_28_NO_6_, the same as garcinamines F and G, suggesting a relationship among these compounds. The ^13^C NMR and DEPT spectra of **3** (see Supplementary Materials) showed twenty carbon atoms but with a pattern that differed from that of garcinamine F. Indeed, it included two methyl groups, seven methylene carbons (all *sp*^3^), six *sp*^3^ methines, and five unprotonated carbon signals (Table 1). The multiplets of the ^1^H NMR spectrum (Table 2) were arranged through the COSY spectrum into three spin systems (Figure 2), evidencing the lack of proton connection between the H_2_-2/H_2_-3 fragment and the remaining protons of the guaiane skeleton. Key HMBC correlations from H_3_-15 to C-3, C-4, and C-5 and from H_2_-2 to C-1 defined the structure of the five-membered ring. Correlations from the allylic H_3_-14 to the oxygenated C-9 and to the *sp*^2^ C-1 and C-10 defined the structure of the seven-membered ring with a double bond between the two latter carbons. Finally, correlations from H-6 to C-12 and from H_2_-13 to the proline carbons C-2′ and C-5′ completed the structure of garcinamine H (**3**). ROESY correlations of garcinamine H paralleled those of garcinamine G for common stereocenters and allowed the assignment of the relative configuration to **3**. Thus, garcinamine H is an isomer of garcinamine G showing a somewhat rare Δ^1,10^ double bond in place of the Δ^10,14^ one. This is the first finding of this structural feature in *A. garcinii*.

**Figure 3:**
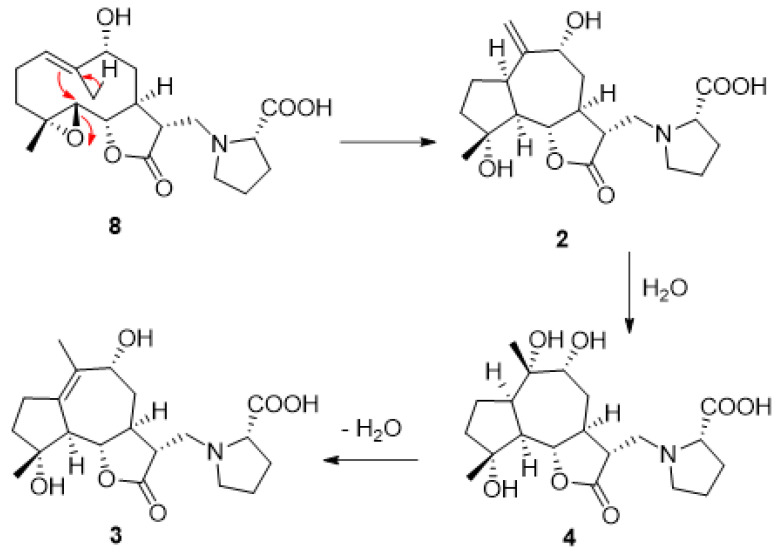
Biosynthetic relationship among some of the isolated sesquiterpenoids.

The sesquiterpenoid derivatives isolated in this study bear an evident biosynthetic relationship. For example, the newly isolated garcinamine G could derive from the epoxide-driven cyclization of the germacranolide garcinamine C. Hydration of the exocyclic double bond of garcinamine G produces garcinamine E that, upon dehydration, would give garcinamine H (**3**) (Figure 3).

### 2.2. Biological Activity

The antiproliferative activity of the isolated compounds **1**–**8** was evaluated on A549 (lung), MCF-7 (breast), and LoVo (colon) carcinoma cell lines using the MTT assay. Table 3 shows IC_50_ values measured after 48 h of treatments. The results evidenced that 9β-hydroxyparthenolide and garcinamines C and D exerted cytotoxic effects in a dose- and time-dependent manner. The activity of 9β-hydroxyparthenolide was not unexpected since it had already been described in the literature [18]. However, better activity was obtained for garcinamine D against A549 cells and thus it was selected for cell cycle analysis by PI staining and apoptosis induction by Annexin-FITC/PI assays.

**Figure 4:**
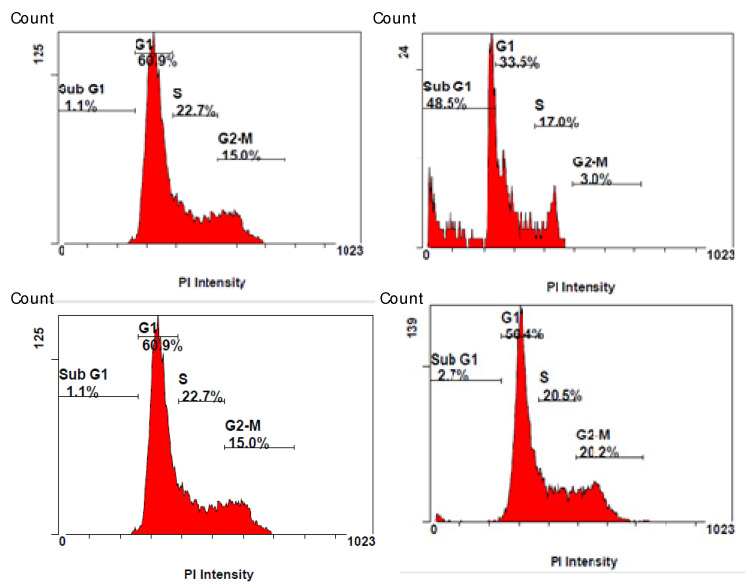
Effect of compounds garcinamine D (**top**) and garcinamine E (**bottom**) on the cell cycle distribution of A549 cells. After treatment with the IC_50_ concentration of garcinamine D (**right**); Untreated (**left**). Distribution of cell cycle phases was performed after 48 h of treatment and was quantitated based on flow cytometric analysis.

The effects of garcinamine D on A549 cell cycle phases were evaluated using PI staining in FACS analysis to quantify the cell percentages in the different phases. For this analysis, A549 cells were treated with garcinamine D (IC_50_ concentration) for 48 h and later stained with PI (Figure 4, top). The results revealed the accumulation of cells in the sub-G1 phase. The number of cells in the sub-G1 phase was 1.1% in the untreated cells, and 48.5% in the treated cells. Furthermore, the cells were treated with one of the non-active compounds (garcinamine E) at the corresponding IC_50_, and as expected, it did not show clear activity (Figure 4, bottom).

**Figure 5:**
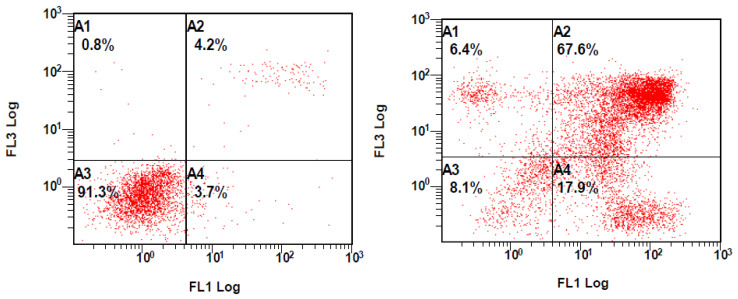
Assessment of apoptosis induction in A549 cells treated with garcinamine D: (**left**) control and (**right**) treated A549 (IC_50_, 48 h). Quarters (A4) represent early apoptosis, (A3) viable cells, (A2) late apoptosis, and (A1) necrosis. The rate of apoptosis was determined by flow cytometry, using the Annexin V-FITC/PI staining assay.

The effect of garcinamine D on the growth inhibition of A549 cells was also investigated by Annexin V/PI assay to show its apoptotic effects. After 48 h of the treatment with the IC_50_ concentration of garcinamine D, 17.9% and 67.6% of cells moved into early and late apoptosis, respectively (Figure 5), demonstrating the apoptosis-inducing effect of garcinamine D. On the other hand, the non-active compound garcinamine E did not show any prominent activity at the corresponding concentration (data not shown).

The close structural similarity within the series of compounds tested for antiproliferative activity can stimulate interesting comments on structure–activity relationships (SARs). Firstly, contrary to other reported cases (see the Introduction), germacrane compounds seemed to be more active than the corresponding guaianes, which is evident if we compare the bioactivities of **7**/**8** with those of the parallel **1**/**2**. Interestingly, although bioactivity of sesquiterpenoid γ-lactones is commonly associated with the presence of an electrophilic exomethylene group [19], compounds **7**/**8** were equally active (or even slightly more active) than the exomethylene-containing 9β-hydroxyparthenolide (**5**). Finally, the amino acid unit included in these adducts appears to play an important role, as evident from the comparison between the inactive valine-bearing garcinamine B (**6**) and the corresponding active proline-bearing garcinamine C (**8**). This observation is not unprecedented, and the antiulcer activity of saussureamines is also modulated by the amino acid unit present in the adduct [20]. In summary, the antiproliferative activity of sesquiterpene-amino acid adducts seems to follow independent SAR compared to simple sesquiterpenoids, and still needs to be studied in detail.

## 3. Materials and Methods

### 3.1. General

The MX-500 Bruker spectrometer (Billerica, MA, USA) was used to measure one-dimensional (1D) and two-dimensional (2D) nuclear magnetic resonance (NMR) spectra. The chemical shifts (δ) were calculated (ppm) relative to TMS, based on the residual solvent signal and *J* scalar coupling constants reported in Hertz (Hz). ESI-MS analyses were carried out on an Agilent (Santa Clara, CA, USA) Triple Quadrupole 6410 QQQ LC/MS mass spectrometer with ESI ion source (60 psi nebulizer pressure, 350 °C gas temperature and 12 L/min flow rate), operating in the negative and positive scan modes of ionization through the direct infusion method using methanol:water (4:6 *v*/*v*) at a flow rate of 0.5 mL/ min. Separations and purifications of secondary metabolites were carried by using column chromatography with either silica gel 70–230 mesh or RP-18 (Merck, Darmstadt, Germany). The TLC plates were RP-18 (Merck) and pre-coated silica gel 60 F_254_, and the spots were detected by UV light and by spraying with ceric sulphate and sulfuric acid reagents followed by heating on a hot plate (TLC plate heater III CAMAG, USA). Dragendorff’s spraying reagent was used to give a dark orange color. Analytical-grade solvents and reagents were obtained from Sigma-Aldrich (St. Louis, MO, USA). NMR deuterated methanol (CD_3_OD) and dimethylsulfoxide (DMSO-*d*_6_) were purchased from Cambridge Isotope Laboratories (Tewksbury, MA, USA).

### 3.2. Plant Material, Extraction and Isolation

*A. garcinii* fresh leaves (1.0 kg) were collected manually in March 2020 from Riyadh city, Saudi Arabia and authenticated by Dr. M. Atiqur Rahman, College of Pharmacy, King Saud University, KSA. A voucher specimen (# 12811) was deposited at the Herbarium of the College of Pharmacy, King Saud University, KSA.

Fresh leaves (1.0 kg) were dried in the shade for up to 2–3 weeks and then crushed into a fine powder. The plant powder was soaked in 80% methanol at room temperature (3 × 15 L), with frequent shaking. The extract was then filtered using filter paper (Whatman No. 1). The filtrate was concentrated under reduced pressure using a rotary evaporator (Büchi Rotavapor RII, Flawil, Switzerland). The obtained crude extract (102.2 g) was suspended in H_2_O (800 mL) and fractionated successively with organic solvent, and the residual water fraction was lyophilized. The *n*-butanol-soluble fraction (75.8 g) was exposed to a silica gel open column and eluted with a gradient of CH_2_Cl_2_:CH_3_OH (9.5:0.5 to 1.0:9.0), to afford eight major fractions (I–VIII) based on their TLC pattern. Fraction I (0.6 g) was isolated at CH_2_Cl_2_:CH_3_OH (9.0:1.0) and further exposed to purification via using normal silica column to give **5** (7.2 mg), CH_2_Cl_2_:CH_3_OH (8.0:2.0). Furthermore, fractions II and III were eluted by using CH_2_Cl_2_:CH_3_OH (8.0:2.0 and 7.5:2.5, respectively) and led to the isolation of compounds **2** and **7** (8.4 mg and 6.9 mg, respectively). From the major silica column, solvent system CH_2_Cl_2_:CH_3_OH (6.5:3.5) gave fraction IV which was further purified by using the RP-C18 column and a mobile phase mixture of water/methanol was used with gradient elution and decreasing polarity to afford compound **4**, 6.0:4.0 H_2_O:MeOH (11.3 mg). Fraction VI was obtained at 6.0:4.0 CH_2_Cl_2_:CH_3_OH gradient elution and further loaded on the RP C-18 column for chromatography, eluted under medium pressure with a gradient of water/methanol (7.5:2.5→9.0:1.0), to obtain two sub-fractions which was further purified by loading on a second RP C-18 column and eluted with a gradient mixture of water/methanol to yield compounds **1** and **3** (9.0 mg and 10.5 mg, respectively). Fraction VII (1.2 g) was eluted with increasing polarity by the addition of methanol to dichloromethane until subfraction VII-a was afforded (4.0:6:0), which was further loaded on an RP-C18 column the elution was continued using gradient polarity reduction, by adding methanol to water gradually, until compounds **6** (9.4 mg) and **8** (12.8 mg) were yielded (4.0:6.0 and 3.0:7.0, H_2_O:MeOH).

Garcinamine F (**1**). Yellow gummy solid; [α]^25^ D-27.2 (c 0.10, CH_3_OH); (+) ESIMS *m/z* 380.2076 [M + H]^+^ (calcd. for C_20_H_30_NO_6_, 380.2073); ^1^H and^13^C NMR data, see Table 1 and Table 2.

Garcinamine G (**2**). Yellow gummy solid; [α]^25^D-22.5 (c 0.15, CH_3_OH); (+) ESIMS *m/z* 380.2080 [M + H]^+^ (calcd. for C_20_H_30_NO_6_, 380.2073); ^1^H and^13^C NMR data, see Table 1 and Table 2.

Garcinamine H (**3**). Yellow gummy solid; [α]^25^D-29.3 (c 0.15, CH_3_OH); (+) ESIMS *m/z* 380.2069 [M + H]^+^ (calcd. for C_20_H_30_NO_6_, 380.2073); ^1^H and^13^C NMR data, see Table 1 and Table 2.

### 3.3. Biological Activity

Cell proliferation assay was carried out using MTT. Three different types of human cancer cell lines (A549 lung carcinoma, MCF-7 breast carcinoma, and LoVo colon carcinoma) were used in this study. The cells were counted and plated in a 24-well cell culture plate with a density of 5 × 10^4^ cells/well and allowed to grow and attach to the plate for 24 h. The compounds were dissolved in dimethyl sulfoxide (DMSO) to prepare stock solution (3 mg/mL). The serial dilutions of compounds (1–30 µM) were added to triplicate wells; mock-treated (0.1% DMSO) cells were used as negative control and doxorubicin was used as positive control. Cells were treated with the appropriate compounds for 48 h. At the end of the treatment period, 1 mL of 5 mg/mL MTT was added to each well, and the plates were further incubated at 37 °C in the presence of 5% CO_2_ for 2–4 h. The media were removed, 0.01 N HCl in isopropanol was added to each well, and the plates were placed in a shaker for 10 min. The absorbance was read at 570 nm using a microplate reader (BioTek, Winooski, VT, USA). The concentration at which cell viability was reduced by 50% (IC_50_) was calculated using Origin Pro 8.5 software.

The antiproliferative effect of compounds **1**–**8** against breast (MCF-7), colon (LoVo), and lung (A549) cell lines were examined using MTT assay. The collected cells were cultured in Dulbecco’s modified Eagle’s medium (DMEM) with 10% heat-inactivated fetal bovine serum, HEPES buffer, 1% L-glutamine, and 50 µg/mL gentamycin, and then placed in 96-well plates at 50,000 cells/well. Compounds were dissolved in DMSO and the cells were treated with different concentrations of each compound for 48 h, and then 3-(4,5-dimethyltiazol-2yl)-2,5 diphenyl tetrazolium bromide (MTT) (5 mg/mL in saline, 10 µL) was added and cells were incubated for an additional 4 h at 37 °C. Thereafter, cells were lysed and dark blue crystals of the formazan product were dissolved with isopropanol and the absorbance intensity was measured at 570 nm using a microplate reader The cell survival percent was calculated as the mean absorbance of treated sample/mean absorbance of control × 100. The dose–response curve of the compounds was analyzed using OriginPro 8.5 software. Doxorubicin was used as a positive control.

Cellular DNA content was analyzed using propidium iodide staining by flow cytometry. In brief, A549 cells were seeded into a 6-well plate in 2 mL culture medium with an IC_50_ concentration and DMSO as a vehicle for 48 h. Thereafter, cells were harvested using trypsin and centrifuged and fixed in 70% ethanol for 4 h at 4 °C. Ethanol-fixed cells were pelleted, washed with ice-cold PBS, and re-suspended in a staining solution containing 50 μg/mL PI and 100 μg/mL RNase. After incubation in the dark for 30 min, the cells were analyzed by flow cytometry.

The apoptotic cells were detected using flow cytometry with the Annexin V-FITC/PI double labeling method. Briefly, after 48 h of treatment with **7** at IC_50_ values, A549 cells were collected, washed and resuspended in Annexin binding buffer. Thereafter, cell staining was performed with FITC and PI (5 µL of each), according to the manufacturer’s instructions (BioLegend, San Diego, CA, USA). Flow cytometric analysis was performed immediately after 20 min of staining. Data acquisition and analysis were performed in a flow cytometer (Cytomics FC 500; Beckman Coulter, Brea, CA, USA).

## 4. Conclusions

Phytochemical investigation of the aerial parts of *Anvillea garcinii* allowed us to expand the knowledge on the sesquiterpenoid composition of this plant with the addition of three new guaianolide-proline adducts named garcinamines F–H. These compounds bear an evident biogenetic relationship with germacranolides, previously isolated from the same plant. All the isolated compounds were evaluated for their antiproliferative activity against three human carcinoma cell lines. Garcinamines C and D and 9β-hydroxyparthenolide exerted cytotoxic effects in a dose- and time-dependent manner. The mechanism of action of garcinamine D was investigated, revealing a marked pro-apoptotic effect with an accumulation of cells in the sub-G1 phase. These results further confirm that plants constitute an immense casket of chemodiversity that can be beneficially used to discover new hits for drug development.

## Figures and Tables

**Table 1:** ^13^C (125 MHz) NMR data for garcinamines F–H (**1**–**3**) in CD_3_OD.

Positions	Garcinamine F (1)	Garcinamine G (2)	Garcinamine H (3)
	δ_H_, type	δ_H_, type	δ_H_, type
1	35.4, CH	35.4, CH	136.2, C
2	26.0, CH_2_	25.6, CH_2_	33.0, CH_2_
3	41.4, CH_2_	41.4, CH_2_	39.2, CH_2_
4	79.5, C	79.6, C	80.3, C
5	55.1, CH	54.9, CH	56.6, CH
6	84.1, CH	84.6, CH	83.3, CH
7	43.0, CH	41.4, CH	47.0, CH
8	39.5, CH_2_	38.5, CH_2_	35.7, CH_2_
9	74.6, CH	73.2, CH	71.4, CH
10	152.6, C	150.8, C	131.3, C
11	41.8, CH	41.6, CH	41.6, CH
12	177.0, C	177.1, C	177.0, C
13	53.0, CH_2_	53.1, CH_2_	52.5, CH_2_
14	108.5, CH_2_	111.1, CH_2_	15.0, CH_3_
15	22.5, CH_3_	22.5, CH_3_	21.8, CH_3_
1′	171.9, C	171.9, C	172.0, C
2′	70.8, CH	70.6, CH	70.6, CH
3′	28.6, CH_2_	28.6, CH_2_	28.6, CH_2_
4′	23.5, CH_2_	23.4, CH_2_	23.4, CH_2_
5′	54.1, CH_2_	54.1, CH_2_	54.2, CH_2_

**Table 2:** ^1^H (500 MHz) NMR data for garcinamines F–H (**1**–**3**) in CD_3_OD.

Positions	Garcinamine F (1)	Garcinamine G (2)	Garcinamine H (3)
	δ_H_ (multiplicity, *J* in Hz)	δ_H_ (multiplicity, *J* in Hz)	δ_H_ (multiplicity, *J* in Hz)
1	3.04 (dd, 4.0, 11.0)	3.56 m	-
2a	1.92 (m)	1.90 (m)	2.12 (m)
2b	1.83, overlapped	1.78 (m)	1.70 (m)
3	1.83–1.85 (m)	1.82 (m)	1.76 (m)
5	2.30 (t, 11.0)	2.28 (t, 11.0)	2.70 (d, 10.5)
6	4.23 (d, 11.0)	4.26 (d, 11.0)	4.02 (bd, 10.5)
7	2.30 (m)	2.52 (m)	2.31 (dd, 3.0, 13.0)
8α	2.39 (dd, 4.0, 9.0)	2.24 (m)	2.06 (d, 11.5)
8β	1.42 (m)	1.65 (m)	1.65 (d, 11.5)
9	3.99 (dd, 4.0, 7.0)	4.56 (bs)	4.23 (bs)
11	3.0 (m)	3.07 (dd, 4.0, 11.5)	3.13 (dd, 3.5, 12.5)
13a	3.63 (dd, 11.0, 12.5)	3.60 (dd, 11.0, 12.5)	3.65 (dd, 10.5, 12.5)
13b	3.53 (dd, 4.0, 12.5)	3.45 (dd, 4.0, 12.5)	3.57 (dd, 3.5, 10.5)
14a	5.51 (bs)	5.15 (bs)	1.77 (s)
14b	5.17 (bs)	5.05 (bs)	-
15	1.33 (s)	1.30 (s)	1.36 (s)
2′	4.02 (dd, 5.0, 9.5)	4.02 (dd, 5.0, 9.5)	4.06 (dd, 6.0, 10.0)
3′a	2.46 (dd, 3.5, 9.5)	2.45 (dd, 3.5, 9.5)	2.46 (dd, 3.5, 10.0)
3′b	2.26 (dd, 5.0, 3.5)	2.24 (dd, 5.0, 3.5)	2.26 (m)
4′a	2.17 (m)	2.15 (m)	2.17 (m)
4′b	1.98 (m)	1.97 (m)	1.99 (m)
5′a	3.86 (dd, 4.0, 7.5)	3.85 (dd, 4.0, 7.5)	3.88 (dd, 4.0, 7.5)
5′b	3.25 (dd, 7.5, 10.5)	3.24 (dd, 7.5, 10.5)	3.27 (dd, 7.5, 10.5)

**Table 3:** Antiproliferative activity (IC_50_ values) of compounds **1**–**8** against different cancer cells ^a^.

Compound	Cell Lines and IC_50_ (µM)		
	A549	LoVo	MCF-7
**1**	NA	NA	NA
**2**	NA	NA	NA
**3**	NA	NA	NA
**4**	NA	NA	NA
**5**	83.7 ± 0.3	121.4 ± 3.2	75.0 ± 5.3
**6**	NA	NA	NA
**7**	31.5 ± 3.1	39.2 ± 3.0	36.0 ± 3.4
**8**	75.8 ± 1.4	38.8 ± 2.1	71.6 ± 5.2
Doxorubicin	0.98 ± 0.02	5.5 ± 0.5	2.3 ± 0.1

^a^ Values are the mean ± SD (*n* = 3); NA = No activity at 100 µM (highest concentration tested). Half maximal inhibitory concentration (IC_50_) values were calculated using OriginPro 8.5 software.

## Data Availability

Not Applicable.

## Supplementary Materials

The following are available online at https://www.mdpi.com/article/10.3390/plants10061130/s1, Figures S1–S18: 1D and 2D NMR spectra of garcinamines F–H.
