# Supplementary material for: Phytochemical Analysis of Anvillea garcinii Leaves: Identification of Garcinamines F–H and Their Antiproliferative Activities
Source: Plants (Basel). 2021 Jun 2;10(6):1130. doi: 10.3390/plants10061130 (PMC8229242; doi:10.3390/plants10061130)
Supplement: Supplementary file 1 [file plants-10-01130-s001.zip › plants-1218147-supplementary.pdf]

SUPPLEMENTARY MATERIAL

Phytochemical Analysis of *Anvillea garcinii* Leaves: Identification of Garcinamines F–H and Their Antiproliferative Activities

Hanan Y. Aati[1], Shagufta Perveen[1*], Raha Orfali[1], Areej M. Al-Taweel[1], Jiangnan Peng[2], Sobia Tabassum[3], Maged S. Abdel-Kader[4], Hasan Soliman Yusufoglu[4] and Orazio Taglialatela-Scafati[5]

[1]Department of Pharmacognosy, College of Pharmacy, King Saud University. P. O. Box 22452, Riyadh 11495, Kingdom of Saudi Arabia;
[2]Department of Chemistry, School of Computer, Mathematical and Natural Sciences, Morgan State University, Baltimore, M D, 21251, USA;
[3]Interdisciplinary Research Centre in Biomedical Materials (IRCBM), COMSATS University Islamabad, Lahore Campus, Pakistan;
[4]Department of Pharmacognosy, College of Pharmacy, Prince Sattam Bin Abdulaziz University, P.O. Box 173, Al-Kharj 11942, Saudi Arabia;
[5]Department of Pharmacy, School of Medicine and Surgery, University of Naples Federico II, Via Montesano 49, 80131 Naples, Italy;

Figure S1. ^{13}C NMR Spectra of Compound **1** in CD_3OD.
Figure S2. DEPT-135 NMR Spectra of Compound **1** in CD_3OD.
Figure S3. ^{1}H NMR Spectra of Compound **1** in CD_3OD.
Figure S4. HSQC Spectra of Compound **1** in CD_3OD.
Figure S5. HMBC Spectra of Compound **1** in CD_3OD.
Figure S6. COSY of Compound **1** in CD_3OD.

Figure S7. ^{13}C NMR Spectra of Compound **2** in CD_3OD.
Figure S8. DEPT-135 NMR Spectra of Compound **2** in CD_3OD.
Figure S9. ^{1}H NMR Spectra of Compound **2** in CD_3OD.
Figure S10. HSQC Spectra of Compound **2** in CD_3OD.
Figure S11. HMBC Spectra of Compound **2** in CD_3OD.
Figure S12. COSY of Compound **2** in CD_3OD.

Figure S13. ^{13}C NMR Spectra of Compound **3** in CD_3OD.
Figure S14. DEPT-135 NMR Spectra of Compound **3** in CD_3OD.
Figure S15. ^{1}H NMR Spectra of Compound **3** in CD_3OD.
Figure S16. HSQC Spectra of Compound **3** in CD_3OD.
Figure S17. HMBC Spectra of Compound **3** in CD_3OD.
Figure S18. COSY of Compound **3** in CD_3OD.

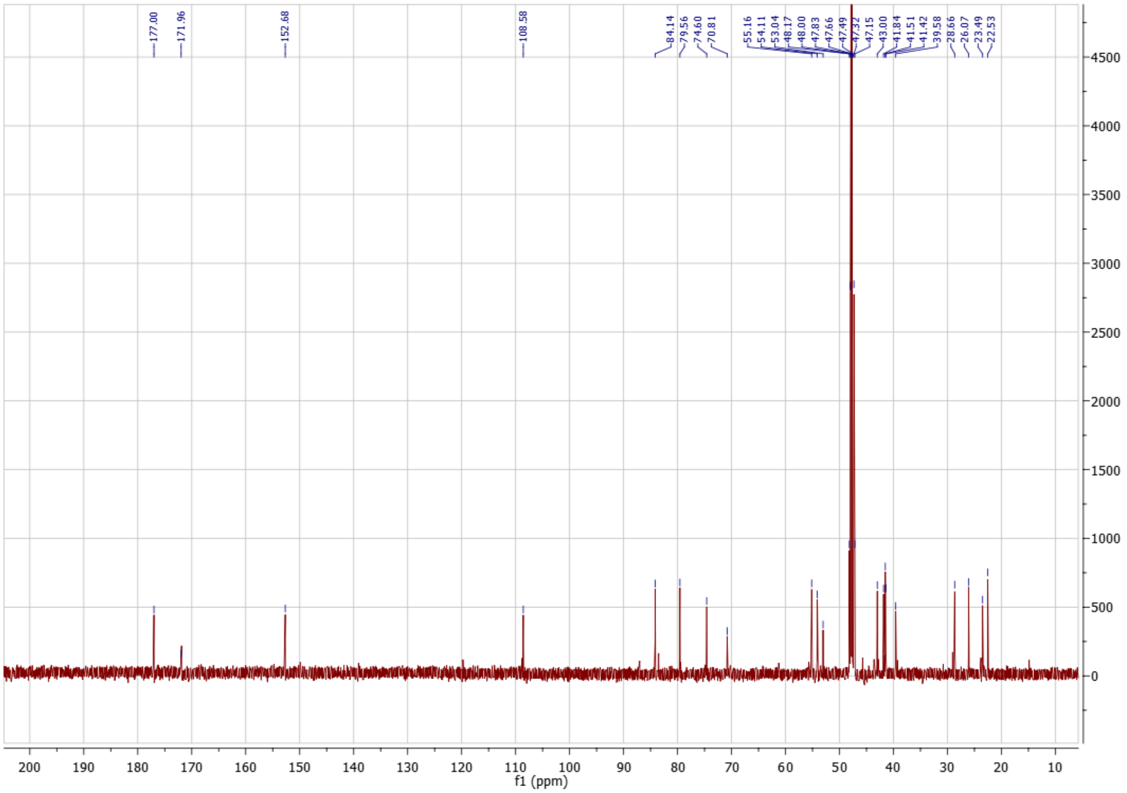

Figure S1. ^{13}C NMR Spectrum of Compound **1** in CD_3OD.

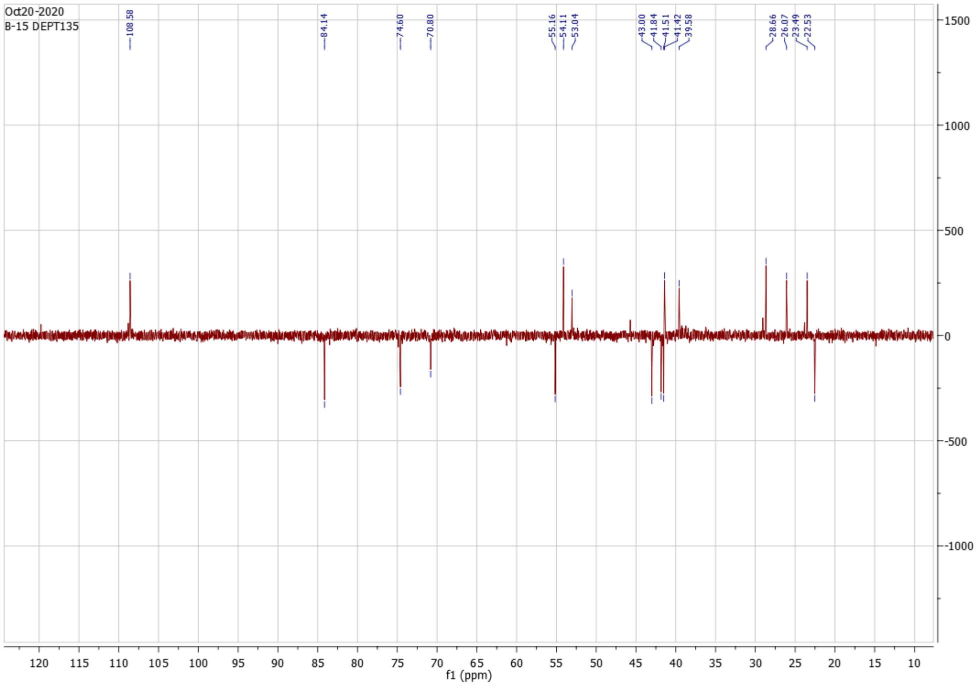

Figure S2. DEPT-135 NMR spectrum of compound **1** in CD_3OD.

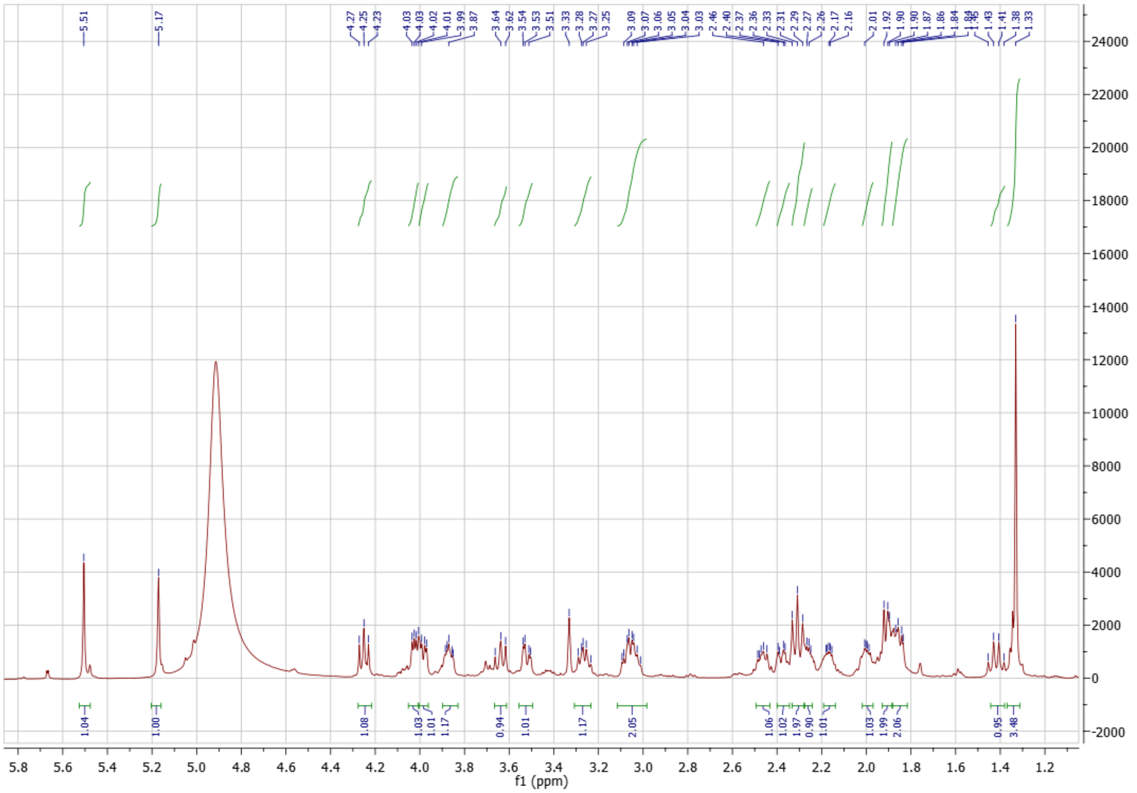

Figure S3. ^{1}H NMR spectrum of compound **1** in CD_3OD.

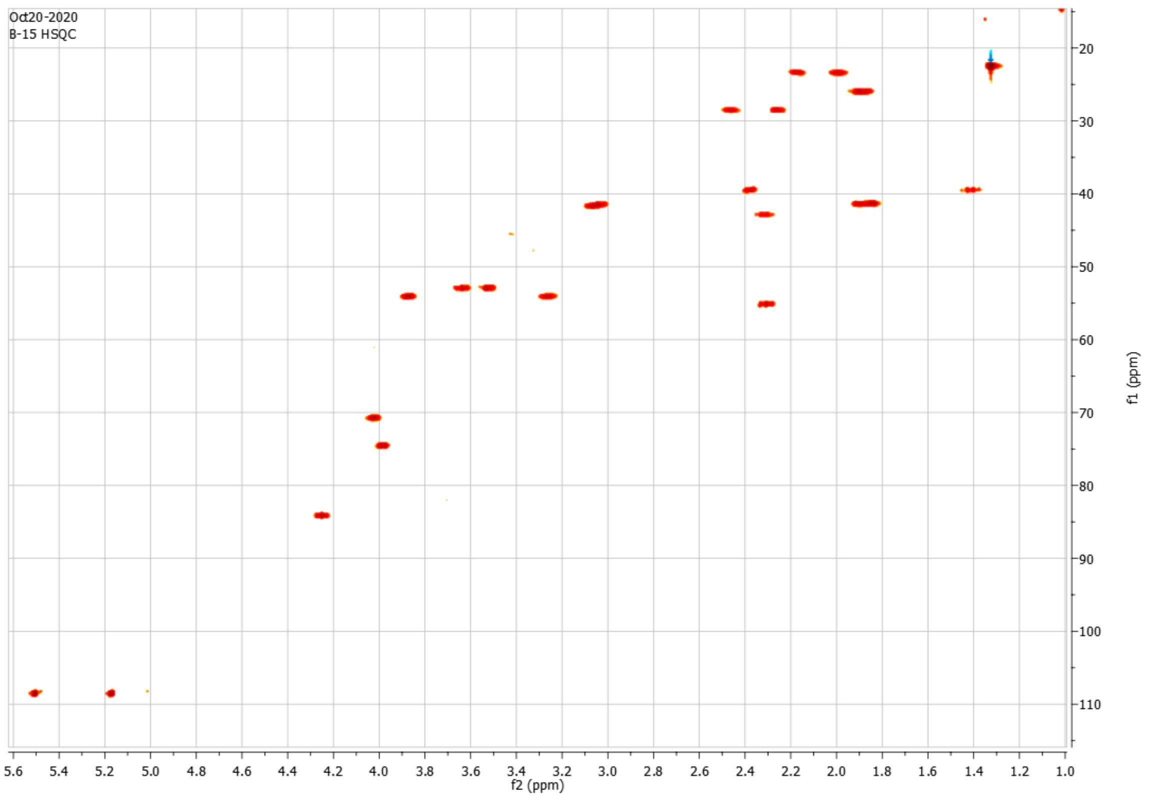

Figure S4. HSQC spectrum of Compound **1** in CD_3OD.

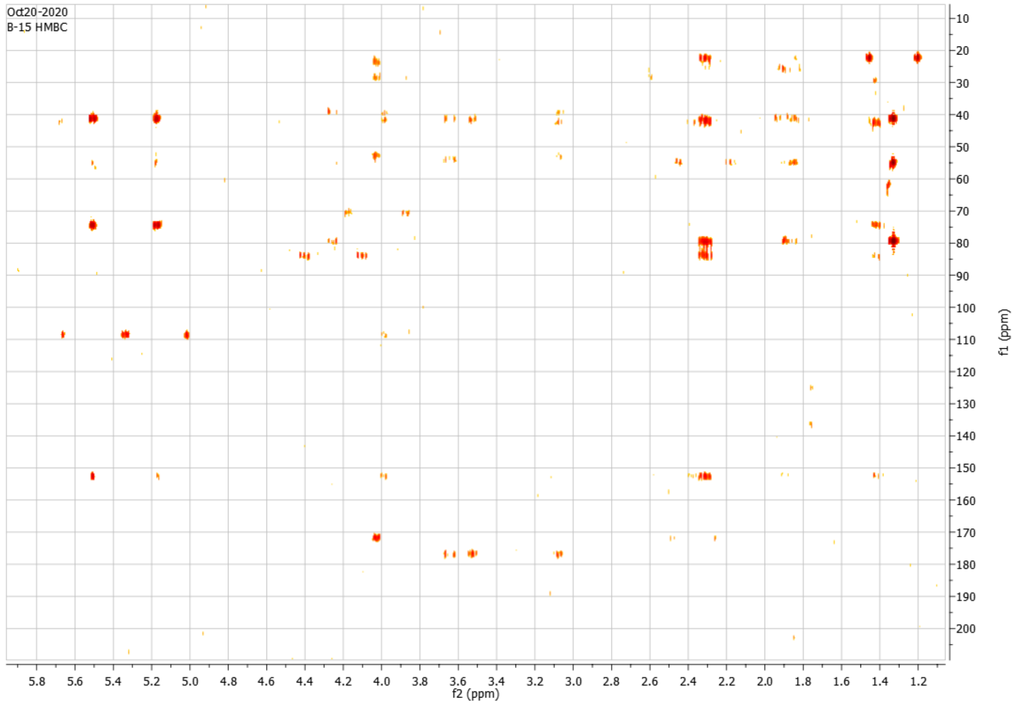

Figure S5. HMBC spectrum of compound **1** in CD_3OD.

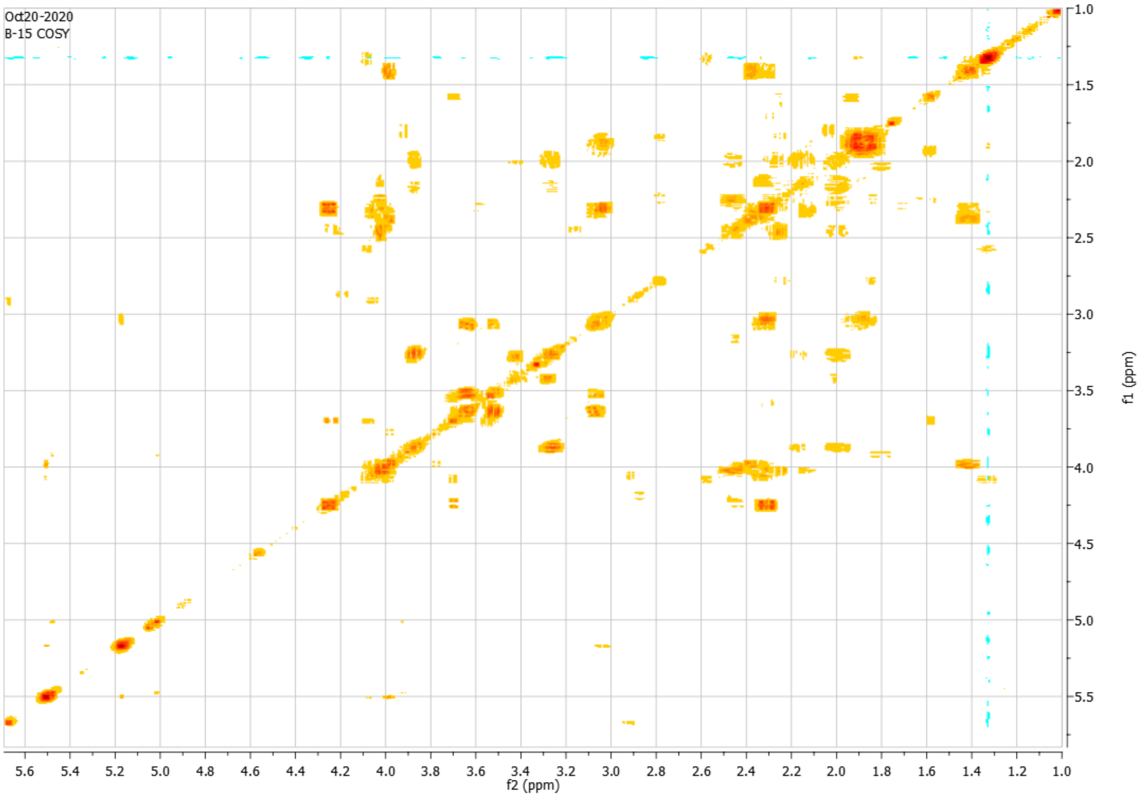

Figure S6. COSY spectrum of compound **1** in CD_3OD.

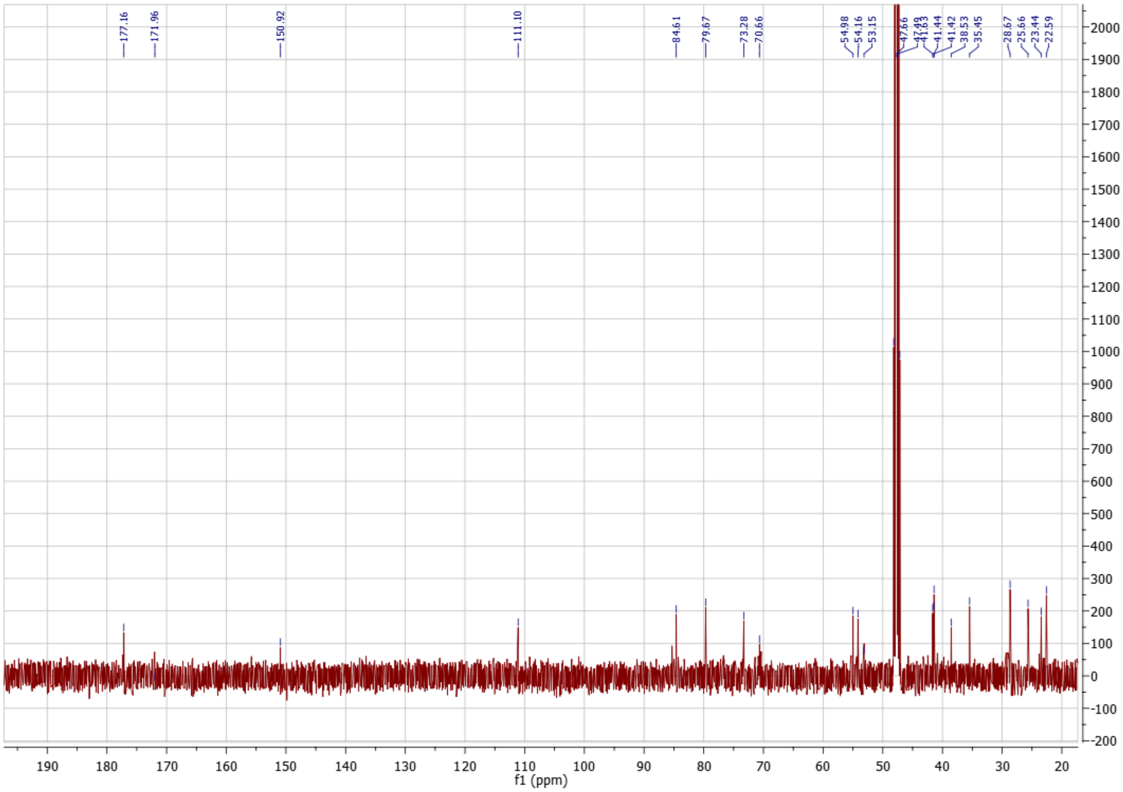

Figure S7. ^{13}C NMR spectrum of compound **2** in CD_3OD.

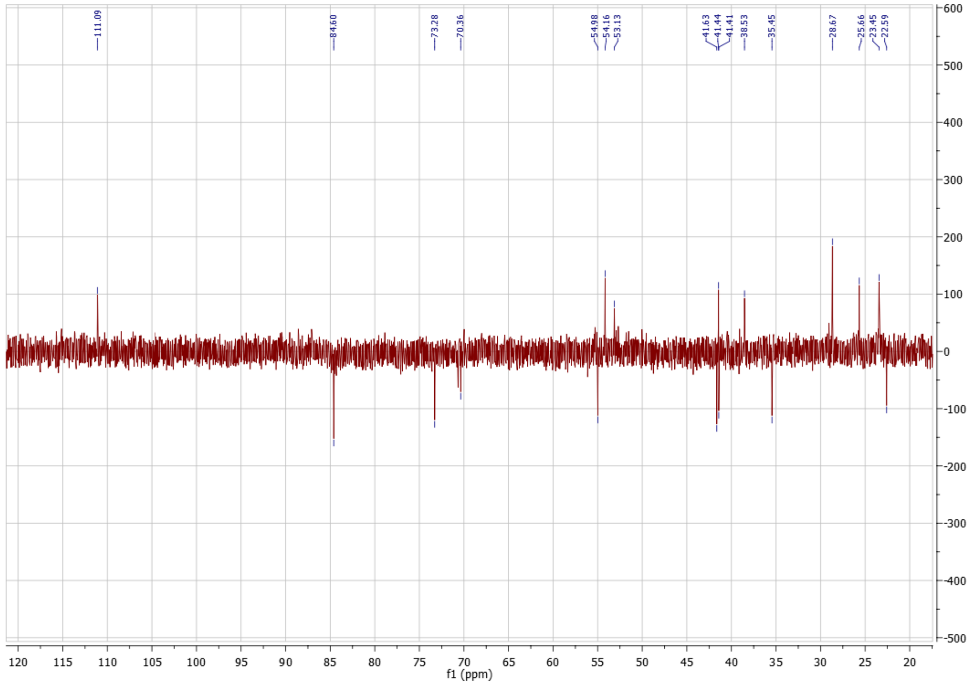

Figure S8. DEPT-135 NMR spectrum of compound **2** in CD_3OD.

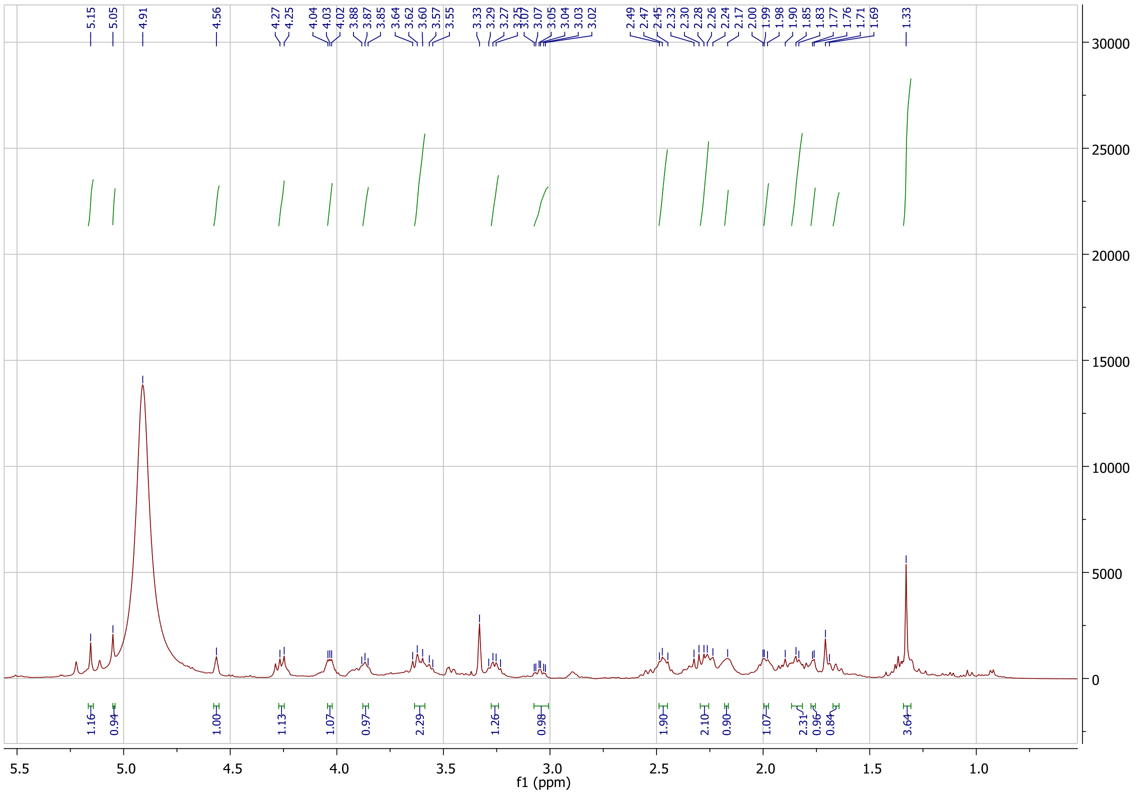

Figure S9. ^{1}H NMR spectrum of compound **2** in CD_3OD.

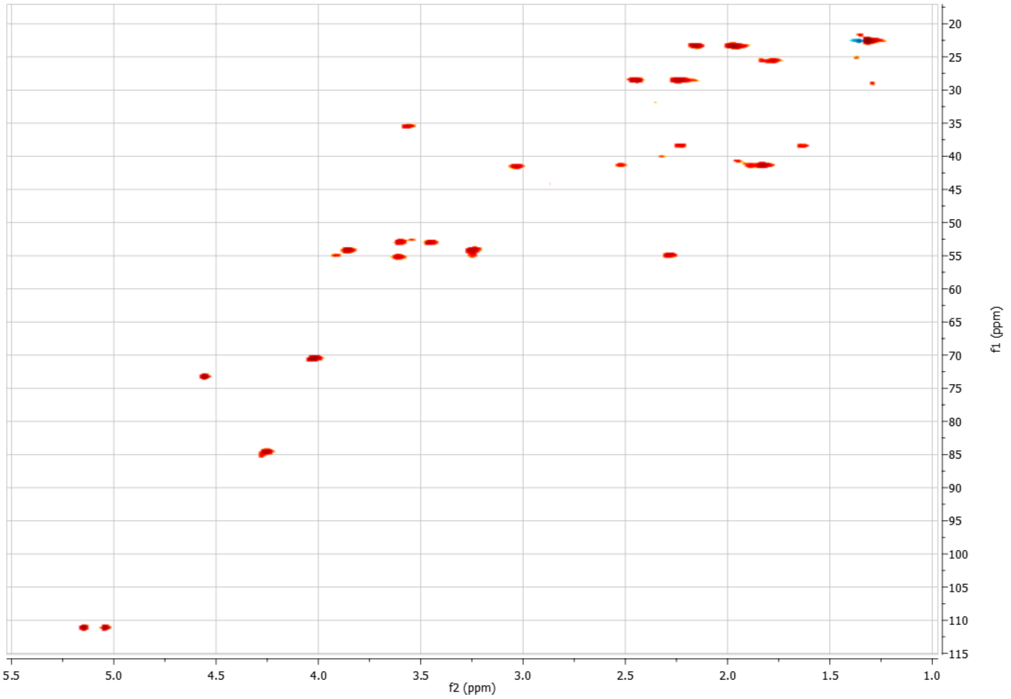

Figure S10. HSQC spectrum of compound **2** in CD_3OD.

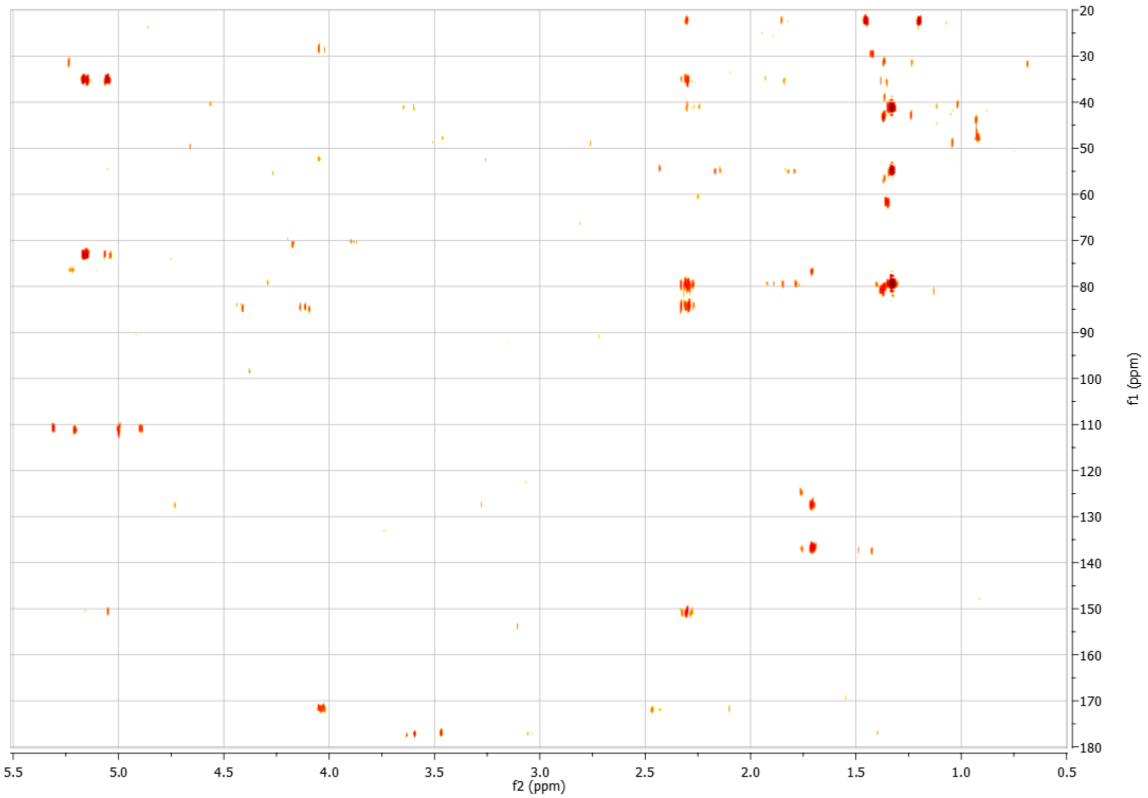

Figure S11. HMBC spectrum of compound **2** in CD_3OD.

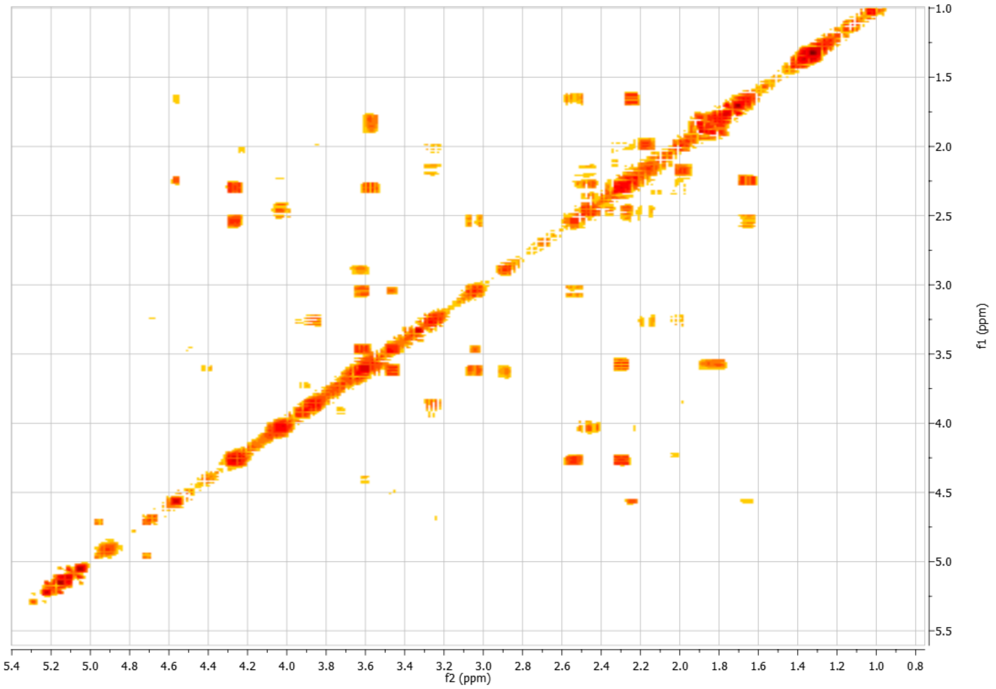

Figure S12. COSY spectrum of compound **2** in CD_3OD.

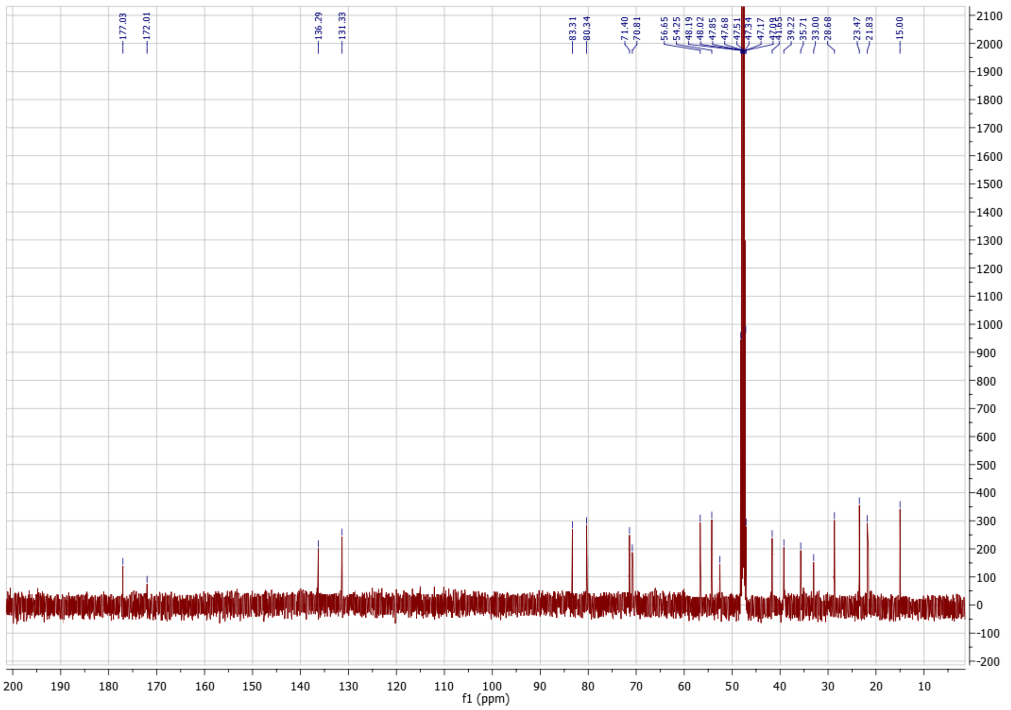

Figure S13. ^{13}C NMR spectrum of compound **3** in CD_3OD.

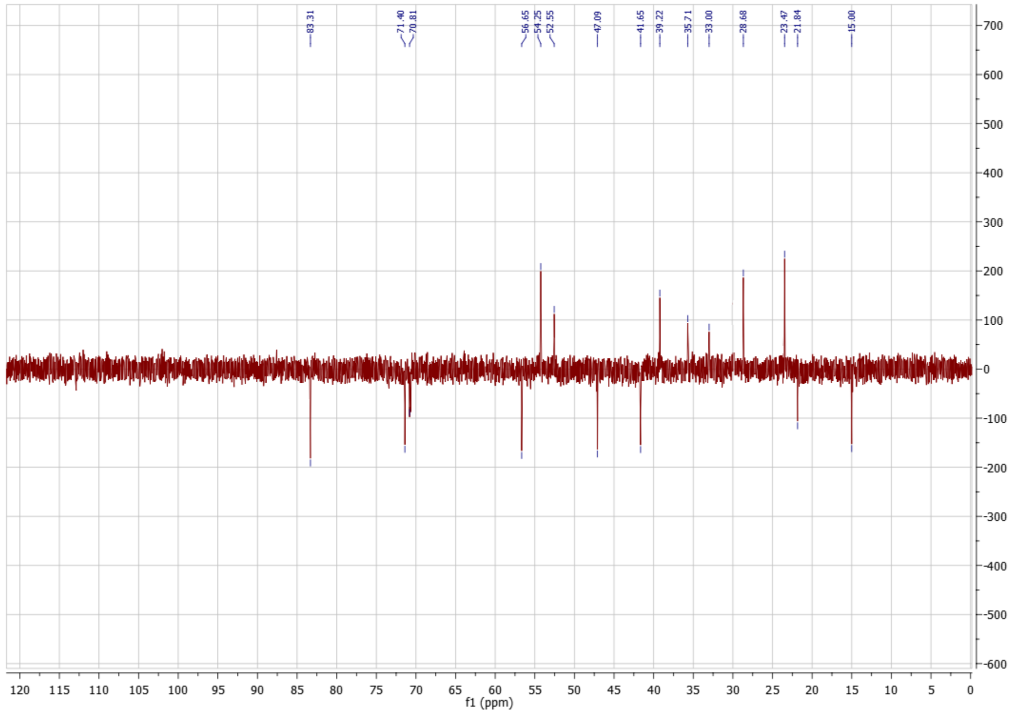

Figure S14. DEPT-135 NMR spectrum of compound **3** in CD_3OD.

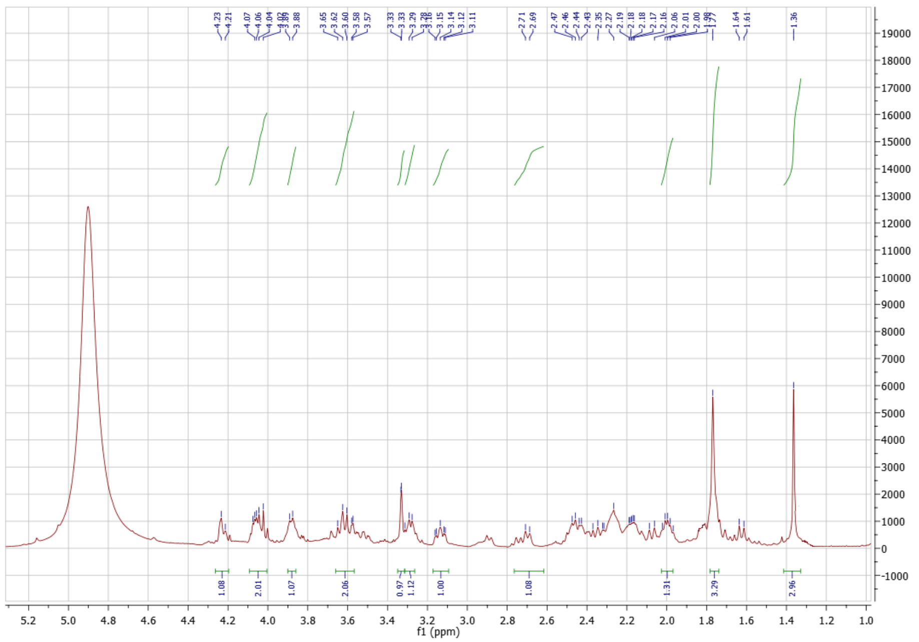

Figure S15. ^{1}H NMR spectrum of compound **3** in CD_3OD.

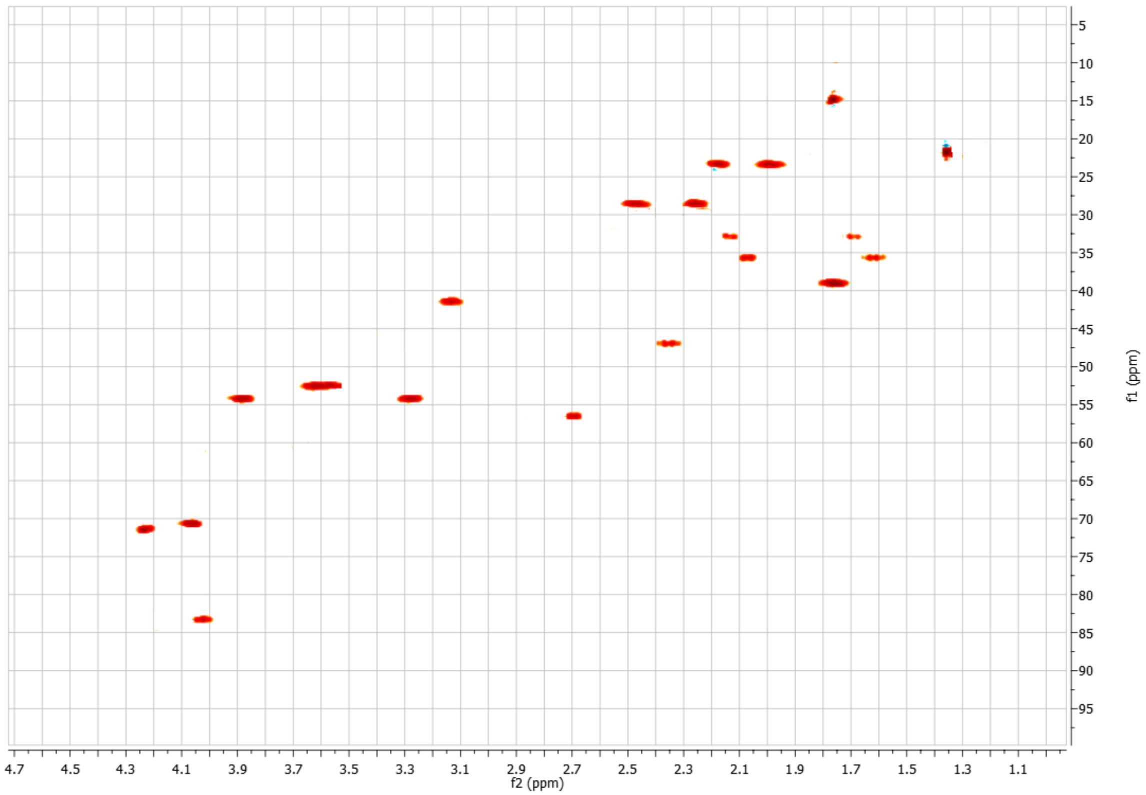

Figure S16. HSQC spectrum of compound **3** in CD_3OD.

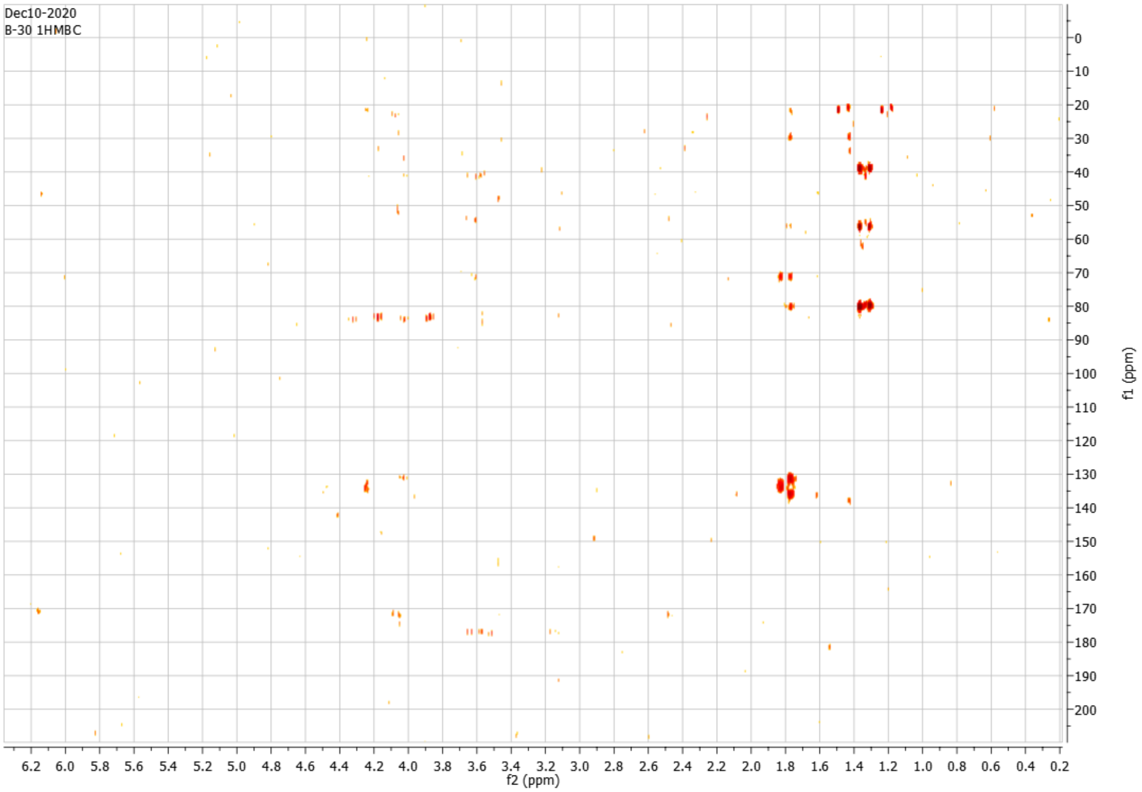

Figure S17. HMBC spectrum of compound **3** in CD_3OD.

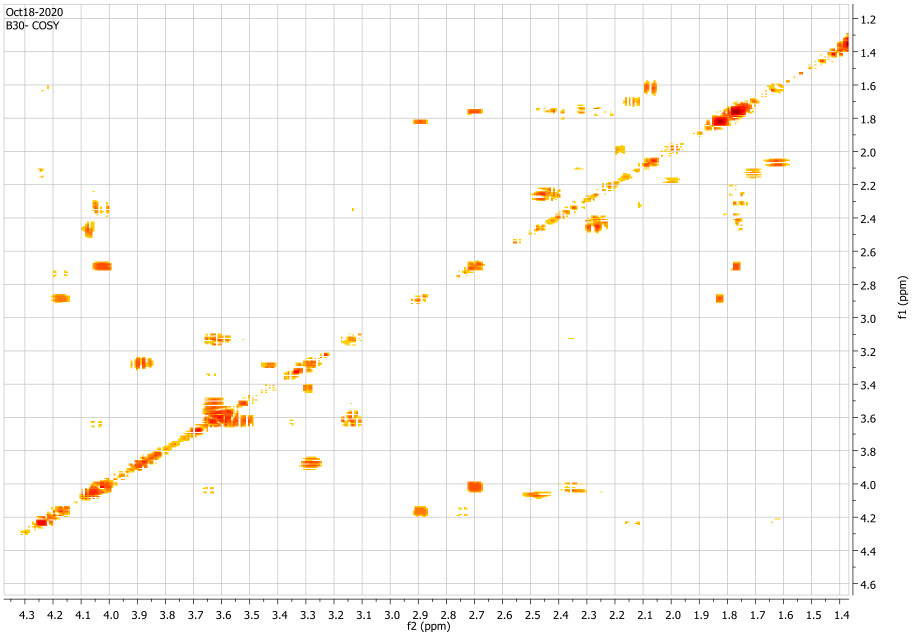

Figure S18. COSY spectrum of compound **3** in CD_3OD.
